# Supplementary material for: Modulating p-AMPK/mTOR Pathway of Mitochondrial Dysfunction Caused by MTERF1 Abnormal Expression in Colorectal Cancer Cells
Source: Int J Mol Sci. 2022 Oct 15;23(20):12354. doi: 10.3390/ijms232012354 (PMC9604058; doi:10.3390/ijms232012354)
Supplement: Supplementary file 1 [file ijms-23-12354-s001.zip › Figure S1.pdf]

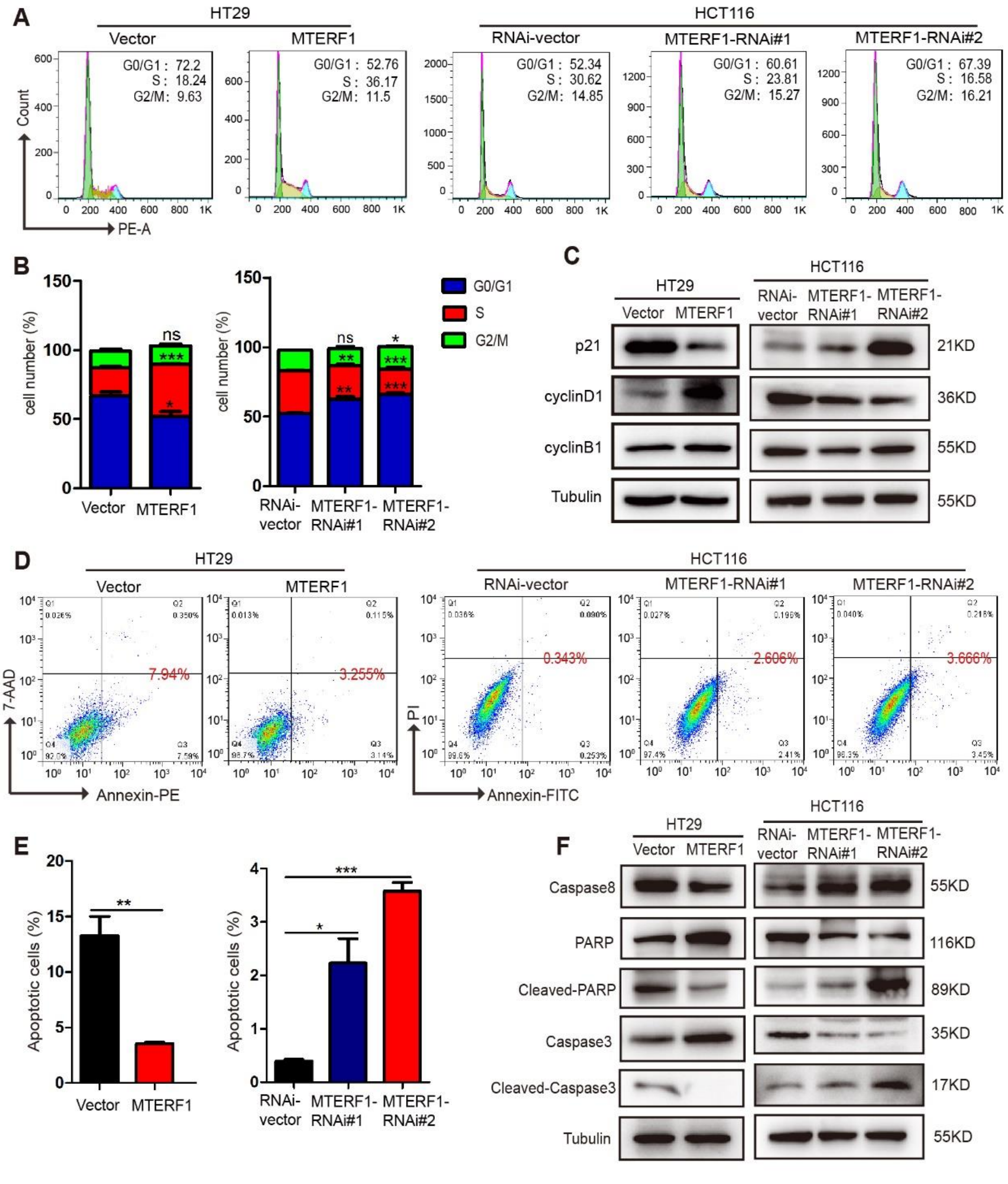

Figure S1. MTERF1 regulates the CRC cell cycle and inhibits CRC cell apoptosis. (A) HT-29 and HCT116 cells were stained with PI to analyze cell cycle distribution upon upregulation and downregulation of MTERF1. (B) Quantification data for (A). (C) Effect of upregulation and downregulation of MTERF1 on the expression of p21, cyclin D1 and cyclin B1 at the protein level in HT-29 and HCT116 cells. α-tubulin was used as an internal control. (D) HT-29 and HCT116 cells were collected for annexin V/PI staining and flow cytometry assays upon upregulation and downregulation of MTERF1. The red number indicates the sum of early and late apoptosis ratios. (E-F) Quantification data and western blotting analysis of the expression levels of apoptosis-related proteins in HT-29 and HCT116 cells upon upregulation and downregulation of MTERF1, including caspase 8, caspase 3 and PARP. α-Tubulin was used as an internal control. Data are presented as

the means ± SEM ($n \geq 3$). NS represents no significant difference. * $P < 0.05$, ** $P < 0.01$, *** $P < 0.001$ by two-tailed Student's t-test.
