# Supplementary material for: Modulating p-AMPK/mTOR Pathway of Mitochondrial Dysfunction Caused by MTERF1 Abnormal Expression in Colorectal Cancer Cells
Source: Int J Mol Sci. 2022 Oct 15;23(20):12354. doi: 10.3390/ijms232012354 (PMC9604058; doi:10.3390/ijms232012354)
Supplement: Supplementary file 1 [file ijms-23-12354-s001.zip › Table S1 Correlation analysis of MTERF1 protein expression with clinicopathological parameters in 52 colorectal cancer patients.pdf]

Table S1. Correlation analysis of MTERF1 protein expression with clinicopathological parameters in 52 colorectal cancer patients (The bold number represents the P-values with significant differences).

Patients characteristics	MTERF1 expression		χ^2	*P*-value
	Low	High		
Age (years)				
＜50	10	10	0.0000, 1	1.0000
≥50	16	16		
Gender				
Female	11	9	0.32501	0.5686
Male	15	17		
Hypertension				
Yes	7	5	0.4331	0.5104
NO	19	21		
Diabetes				
Yes	1	4	1.9911	0.1582
NO	25	22		
Tumor size				
＜5cm	17	11	2.7861	0.0951
≥5cm	9	15		
Lymph node status				
0	5	15	**8.1251**	**0.0044**
1-3	21	11		
ER expression				
Positive	11	5	3.2501	0.0714
Negative	15	21		
PR expression				
Positive	12	15	0.69331	0.4050
Negative	14	11		
Tumor status				
T1+T2	21	10	**9.6651**	**0.0019**
T3+T4	5	16		
Nodal status				
N0	5	15	**8.1251**	**0.0044**
Nx	21	11		
Clinical stages				
1	21	11	**8.1251**	**0.0044**
2	5	15		
