# Supplementary material for: Modulating p-AMPK/mTOR Pathway of Mitochondrial Dysfunction Caused by MTERF1 Abnormal Expression in Colorectal Cancer Cells
Source: Int J Mol Sci. 2022 Oct 15;23(20):12354. doi: 10.3390/ijms232012354 (PMC9604058; doi:10.3390/ijms232012354)
Supplement: Supplementary file 1 [file ijms-23-12354-s001.zip › Table S2 Sequences of primers.pdf]

Table S2. Sequences of primers used in qPCR

| Gene | Primer | Nucleotide sequences |
|---|---|---|
| *GAPDH* | F | 5'-ACAACTTTGGTATCGTGGAAGG-3' |
| | R | 5'-GCCATCACGCCACAGTTTC-3' |
| *ND1* | F | 5'-ATGGCCAACCTCCTACTCCT-3' |
| | R | 5'-GCGGTGATGTAGAGGGTGAT-3' |
| *ND6* | F | 5'-CCACAGCACCAATCCTACCT-3' |
| | R | 5'-TGATTGTTAGCGGTGTGGTC-3' |
| *TFAM* | F | 5'-GGCACAGGAAACCAGTTAGG-3' |
| | R | 5'-CAGAACACCGTGGCTTCTAC-3' |
| *Cytb* | F | 5'-AACCGCCTTTTCATCAATCG-3' |
| | R | 5'-TAGCGGATGATTCAGCCATAATT-3' |
| *12S rRNA* | F | 5'-AAACTGCTCGCCAGAACACT-3' |
| | R | 5'-CATGGGCTACACCTTGACCT-3' |
| *16S rRNA* | F | 5'-CACTGTCAACCCAACACAGG-3' |
| | R | 5'-GGCAGGTCAATTTCACTGGT-3 |
| *NDUFB8* | F | 5'-CCGCCAAGAAGTATAATATGCGT-3' |
| | R | 5'-TATCCACACGGTTCCTGTTGT-3' |
| *18S rDNA* | F | 5'- CGCGCTCTACCTTACCTACC-3' |
| | R | 5'- CCGTCGGCATGTATTAGCTC-3' |
| *D-loop* | F | 5'- GGGAACGTGTGGGCTATTTA-3' |
| | R | 5'- TACTCAAATGGGCCTGTCCT-3' |
